# Does the aldosterone: renin ratio predict the efficacy of spironolactone over bendroflumethiazide in hypertension? A clinical trial protocol for RENALDO (RENin-ALDOsterone) study

**DOI:** 10.1186/1471-2261-7-14

**Published:** 2007-05-09

**Authors:** Hari K Parthasarathy, Khamis Alhashmi, Alex D McMahon, Allan D Struthers, John MC Connell, Gordon T McInnes, Ian Ford, Thomas M MacDonald

**Affiliations:** 1 Division of Medicine & Therapeutics, Ninewells Hospital & Medical School, University of Dundee, Dundee DD1 9SY, UK; 2 Division of Cardiovascular and Medical Sciences, Gardiner Institute, Western Infirmary, 44 Church Street, Glasgow G11 6NT, UK; 3 Robertson Centre for Biostatistics, University of Glasgow, Boyd Orr Building, Glasgow G12 8QQ, UK

## Abstract

**Background:**

High blood pressure is an important determinant of cardiovascular disease risk. Treated hypertensives do not attain a risk level equivalent to normotensives. This may be a consequence of suboptimal blood pressure control to which indiscriminate use of antihypertensive drugs may contribute. Indeed the recent ALLHAT[1]study suggests that thiazides should be given first to virtually all hypertensives. Whether this is correct or whether different antihypertensive therapies should be targeted towards different patients is a major unresolved issue, which we address in this study.

The measurement of the ratio of aldosterone: renin is used to identify hypertensive subjects who may respond well to treatment with the aldosterone antagonist spironolactone. It is not known if subjects with a high ratio have aldosteronism or aldosterone-sensitive hypertension is debated but it is important to know whether spironolactone is superior to other diuretics such as bendroflumethiazide in this setting.

**Methods/design:**

The study is a double-blind, randomised, crossover, controlled trial that will randomise 120 hypertensive subjects to 12 weeks treatment with spironolactone 50 mg once daily and 12 weeks treatment with bendroflumethiazide 2.5 mg once daily. The 2 treatment periods are separated by a 2-week washout period. Randomisation is stratified by aldosterone: renin ratio to include equal numbers of subjects with high and low aldosterone: renin ratios.

Primary Objective – To test the hypothesis that the aldosterone: renin ratio predicts the antihypertensive response to spironolactone, specifically that the effect of spironolactone 50 mg is greater than that of bendroflumethiazide 2.5 mg in hypertensive subjects with high aldosterone: renin ratios.

Secondary Objectives – To determine whether bendroflumethiazide induces adverse metabolic abnormalities, especially in subjects with high aldosterone: renin ratios and if baseline renin measurement predicts the antihypertensive response to spironolactone and/or bendrofluazide

**Discussion:**

The numerous deleterious effects of hypertension dictate the need for a systematic approach for its treatment. In spite of various therapies, resistant hypertension is widely prevalent. Among various factors, primary aldosteronism is an important cause of resistant hypertension and is now more commonly recognised. More significantly, hypertensives with primary aldosteronism are also exposed to various other deleterious effects of excess aldosterone. Hence treating hypertension with specific aldosterone antagonists may be a better approach in this group of patients. It may lead on to better blood pressures with fewer medications.

## Background

### Epidemiology

One approach to more effective antihypertensive control would be to target aldosterone antagonists towards patients in whom these drugs are likely to work best. Hyperaldosteronism is characterised by excessive excretion of aldosterone with concomitant suppression of renin associated with hypertension. However, neither aldosterone excretion nor plasma renin activity alone has proved useful in screening for this condition for a variety of reasons. Hiramatsu and colleagues have suggested that the aldosterone: renin ratio may be a useful screening test[2]. Using the aldosterone: renin ratio to find possible cases, Gordon and colleagues reported that hyperaldosteronism was more common than suspected, with 8% of cases referred to his clinic having this condition[3]. A high aldosterone: renin ratio has been found in 15% of a UK hypertension clinic[4] and the general community[5]. There is debate as to whether a raised ratio defines aldosteronism or whether it detects a subgroup of subjects with aldosterone-sensitive hypertension, although subjects with a raised ratio do appear to have genetic differences from those who do not[6]. If further testing is undertaken, 94% of clinic subjects with a raised ratio did not suppress plasma aldosterone with salt loading, a test many regard as diagnostic[4]. However, salt-loading is not without risks[7] and a simple blood test that was able to guide appropriate therapy is an attractive concept.

### Rationale for study

Most subjects with a raised aldosterone: renin ratio do not have distinct metabolic abnormalities. Furthermore, if they are investigated, most do not have adrenal adenomas; instead they have adrenal hyperplasia or even normal sized adrenal glands. While some authorities recommend full investigation and laparoscopic adrenalectomy if excessive aldosterone secretion can be lateralised to one of the glands[8,9], surgery is not without risks and 60% of adrenalectomised subjects require antihypertensive therapy[10]. In addition, those who have a good response to surgery also show a good response to spironolactone[11] and long term drug treatment is safe and effective[12]. Furthermore, there remains some debate about the true distinction between genuine autonomous primary aldosteronism and relative aldosterone excess association with low renin hypertension. The purpose of the proposed study is not to resolve this argument, but instead to answer a pragmatic question about the best treatment of patients with resistant hypertension. There is no argument that patients with a high aldosterone: renin ratio may have resistant hypertension. It is also clear that the number of patients with a high ratio forms a large sub-group of patients with high blood pressure. Aldosterone has deleterious effects on the cardiovascular system and it is possible that patients with hypertension who have a high aldosterone: renin ratio are at particular risk of cardiovascular morbid events. Thus, it is of considerable practical importance to define what treatment provides best blood pressure lowering and cardiovascular protection to these subjects [13-15] In this regard, a recent observational study done by one of the authors provides some evidence that subjects with a raised aldosterone: renin ratio had good anti-hypertensive responses to more usual doses of spironolactone (25–100 mg)[16]. Importantly, the subjects treated with spironolactone had better blood pressure control with fewer antihypertensive drug treatments after spironolactone was commenced.

There is on going debate on the topic of the aldosterone: renin ratio and its role, if any, in subjects with hypertension. It is true that we need better evidence of the practical value of measuring the ratio before we recommend change to current practice and treat patients with spironolactone. A key question is "would such a strategy be better than treating all subjects conventionally with a thiazide diuretic?"

### Rationale for doses of bendroflumethiazide and spironolactone

Previous studies with thiazide diuretics have suggested that the dose-response relationship is shallow. With bendroflumethiazide, the most commonly used thiazide in the UK, 2.5 mg is almost as effective as 5 mg and as a consequence very few physicians use the higher dose. In essential hypertension, a similar dose-response relationship is true for spironolactone but higher doses of spironolactone may reduce BP more in those with high aldosterone: renin ratios. In one sense, the ideal comparison would be between exactly equipotent doses of these two drugs. However, the critical question is whether the efficacy of the usually prescribed doses of these drugs is predicted by the aldosterone: renin ratio. This is the pragmatic question that is relevant to day-to-day clinical practice. In order to test this hypothesis, it is not necessary for the doses employed to be exactly equipotent. For this reason we have chosen 50 mg of spironolactone, to be compared with 2.5 mg of bendroflumethiazide, the most commonly used doses of each drug.

### Rationale for choice of subjects

In an ideal study, subjects would be untreated at entry. However, patients with high aldosterone: renin ratios tend to have resistant hypertension. For ethical reasons, it is not possible to discontinue anti-hypertensive therapy in such individuals. In addition, in a pragmatic study allowing drug treatment at entry will result in the study population being more representative of the true patient population. We recognise that it is necessary to restrict the types of drug therapy used for the duration of the trial to maximise the information gained from the randomised arms. Although some drugs may independently affect renin and aldosterone, in a crossover trial these effects should be balanced. Thus, subjects taking medication at screening continue on or are changed to standard open drug therapy to control blood pressure in the following order of preference: alpha-blockers, centrally acting drugs, calcium channel blockers, cardio-selective beta blockers and (exceptionally) ACE inhibitors and angiotensin receptor blockers. Allowing subjects into the study who are hypertensive on drug therapy enriches the study population with cases of resistant hypertension. We accept that this may bias the study towards a secondary care type of population, but we believe that many such patients do exist in primary care, albeit in smaller numbers and that the study will still be relevant to that setting.

## Methods/design

### Study design

The study is a double-blind, randomised, crossover, controlled trial that will randomise 120 hypertensive subjects to 12 weeks treatment with spironolactone 50 mg once daily and 12 weeks treatment with bendroflumethiazide 2.5 mg once daily. The 2 treatment periods are separated by a 2-week washout period. Investigators and subjects do not know the order of the treatment periods, which is according to a computer generated randomisation list. Randomisation is stratified by aldosterone: renin ratio to include equal numbers of subjects with high and low aldosterone: renin ratios. This is necessary as in an unselected population, only 15% of subjects will have an aldosterone: renin ratio > 750.

### Study population

Potential study subjects with mild-moderate hypertension are identified from clinic patients or from community treated patients.

### Inclusion/exclusion criteria

#### Screening

Written informed consent is obtained prior to carrying out screening procedures. Potential subjects are assigned a subject number. Antihypertensive therapy may not be necessarily be discontinued[17] but may be in some cases, or it may be adjusted if necessary. Specifically, potential subjects on spironolactone, and for whom, in the opinion of the investigator, there is no compelling indication not to discontinue this for the purpose of taking part in the study, may have this drug discontinued 3 months prior to entry, provided full written informed consent is obtained. Diuretics should be discontinued for a minimum of 2 weeks prior to entry and also beta-blockers, where possible. Alternative antihypertensive treatment may be substituted.

Medical history, concomitant conditions and medications are recorded and a physical examination carried out. Height and weight are measured. Blood pressure is measured using the OMRON 705CP[18]. The mean of three readings is taken after two minutes rest in the sitting position with the arm supported at the level of the heart. A 12-lead ECG and clinical laboratory tests including urinalysis, blood biochemistry and haematology are carried out. Blood samples for measurement of plasma aldosterone and plasma renin activity are taken (after 5 min sitting quietly).

Subjects undergo ambulatory blood pressure monitoring for 26 hours using Spacelabs 90207 monitor. This is set to take readings every 15 minutes during daytime (0800 h–2159 h) and every 30 minutes during night time (2200 h–0759 h). To be considered evaluable, the ABPM report must contain at least 24 hours data, including at least one valid reading for every hour during daytime and every 2 hours during night time. To fulfil the inclusion criteria, mean daytime systolic BP on screening ABPM must be at least 140 mmHg.

#### Randomisation

Blood samples for aldosterone and renin, from subjects who fulfil all other inclusion criteria, are analysed by a central laboratory.

#### Stratified randomisation

The aldosterone: renin ratio calculated by the central laboratory is used to stratify subjects. For the purposes of this study subjects must have either a high aldosterone: renin ratio > 750 with a plasma aldosterone > 250 pmol/l or a low aldosterone: ratio < 300 with plasma renin activity <10 ng/ml/h.

#### Follow-up visits

Randomised subjects are seen at one week, four weeks, eight weeks and twelve weeks during each treatment phase. On each occasion blood pressure is measured using OMRON 705CP and a safety blood sample taken for potassium, magnesium and creatinine. Subjects are also asked if they have experienced adverse events or any changes in concomitant medication. Investigators may repeat ambulatory blood pressure monitoring at follow-up visits if they wish to. These data will not be used in the analysis.

On the penultimate day of each treatment period subjects have Omron BP measurements prior to fitting an ambulatory blood pressure monitor. On their return 26 hours later, on their final day of treatment, subjects again have Omron BP measurements. The mean of these two mean blood pressures is a secondary endpoint of the study. Mean daytime ambulatory blood pressure and mean night time blood pressure are also secondary blood pressure endpoints. The primary endpoint is mean 24-hour blood pressure.

At the end of the study, subjects are commenced or continued on standard anti-hypertensive therapy and followed up as per usual clinical care.

### Safety considerations

#### Assessments

Safety assessments consist of monitoring and recording all adverse events (AEs) and serious adverse events (SAEs), measurement of blood pressure and heart rate, monitoring of blood biochemistry, ECG recordings and physical examination if indicated.

#### Uncontrolled blood pressure

If blood pressure is uncontrolled investigators may use their discretion and add further antihypertensive medication in the following order of preference: alpha-blockers, centrally acting drugs, calcium channel blockers, cardio-selective beta blockers and (exceptionally) ACE inhibitors and angiotensin receptor blockers. A log of all medication taken during the study is kept for each subject.

#### Symptomatic hypotension

If the investigator suspects that a subject is suffering symptomatic hypotension, then open-label anti-hypertensive medication can be withdrawn or study medication can be reduced to one capsule every second day.

#### Hypokalaemia/hyperkalaemia

Subjects who develop hypokalaemia (K^+ ^< 3.5 mmol/l) are given slow release potassium supplementation 600 mg three times daily. If hypokalaemia is deemed severe (K^+ ^< 3 mmol/l) study medication is discontinued for three days or until K^+ ^is > 3.5 mmol/l whereupon study medication is restarted at one capsule taken every second day along with potassium supplementation. Subjects who subsequently develop hypokalaemia have study medication withdrawn. Similarly, study medication is stopped temporarily and restarted at half dosage if plasma K^+ ^> 5.5 mmol/l.

### Endpoints

#### Primary endpoint

The primary endpoint is the difference in mean 24-hour systolic ambulatory blood pressure recorded at the end of each 12-week treatment period.

#### Secondary endpoints

Secondary endpoints include the differences between the following measurements taken at the end of each 12-week treatment period

• Mean 24 hour diastolic ambulatory blood pressure

• Mean daytime ambulatory blood pressure

• Mean night time ambulatory blood pressure

• Mean clinic blood pressure defined as mean of mean clinic BPs on both penultimate and final days of treatment periods

• Clinical biochemistry measurements of plasma potassium (K^+^), magnesium (Mg^2+^), creatinine, triglycerides, cholesterol and HDL cholesterol

### Statistical analysis

#### Power calculations and sample size

Power calculations are based on a standard deviation of 11 mmHg for ambulatory systolic BP and 7 mmHg for ambulatory diastolic BP. A sample size of 28 in each stratum of aldosterone: renin ratio would give 80% power to detect a 6 mmHg difference in ambulatory systolic BP and 4 mmHg difference in diastolic BP between spironolactone and bendroflumethiazide treatments. However, we are interested in detecting differences in BP changes comparing the low-ratio and high-ratio arms. We therefore need to randomise 54 subjects into each arm to detect a 6/4 mmHg difference in BP at the α = p < 0.05 (two-sided). Therefore, allowing for dropouts, a sample size of 60 in each arm or 120 subjects in total are required. A multivariate analysis to investigate the effect of age and other characteristics on the effects of Spironolactone is also planned.

#### Analyses

For each outcome the measurement from the spironolactone treatment period will be subtracted from that of the bendroflumethiazide treatment period. Therefore each person has an estimated "treatment effect". The mean difference, between the treatment effects in the high ratio group and those in the low ratio group, will be compared by t-tests and 95% confidence intervals. There may be some secondary Normal Linear Models that also examine other factors, which may also be considered to be confounders, as this is a non-randomised comparison.

The second analysis will be by conventional crossover analyses of the efficacy of spironolactone (in the high-ratio sub-group only). These analyses will be carried out by Normal Linear Models, that include parameters for patient, period and treatment. In the event that a variable is not normally distributed then the within-patient treatment differences will be calculated and then analysed by either t-tests or Wilcoxon tests.

## Substudies

The reason for these sub-studies is that the indices measured (QT dispersion, heart rate variability, endothelial function and exercise BP) are considered reasonably good surrogates for future cardiovascular events and mortality [19-21] Certainly, in the case of chronic heart failure, the spironolactone-induced improvement in mortality in Randomised Aldactone Evaluation Study (RALES) [22] was paralleled by spironolactone induced improvements in QT dispersion, heart rate variability[23] and endothelial function[24]. The results of these substudies may be useful indicators of future cardiovascular outcomes.

### QT dispersion

QT dispersion is measured from routine ECGs recorded at baseline and at the end of each treatment period. Spironolactone has been shown to reduce QT dispersion in chronic heart failure[23] and it is of interest to investigate whether this is also the case in hypertension and whether such an effect is more prominent in subjects with a high aldosterone: renin ratio.

### Heart rate variability

Heart rate variability is assessed from 24-hour ambulatory ECG monitoring carried out at the end of each treatment phase. Again, spironolactone has been shown to improve the parasympathetic component of heart rate variability[23] and it is of interest to examine if this is also the case in hypertension and whether such an effect is more prominent in subjects with a high aldosterone: renin ratio. Ventricular ectopic activity on the 24 hr tapes is also measured.

### Endothelial function

A subset of the subjects undergoes endothelial function assessment by a standard protocol. At the end of each treatment period, these subjects undergo a detailed brachial artery infusion study with sequential infusions of acetylcholine, nitroprusside and LNMMA (NG-monomethyl-L-arginine) combined. In CHF, spironolactone improves endothelial function[24] even in the presence of an ACE inhibitor. It will therefore be of major interest to see the effect of spironolactone in hypertension and whether such an effect is more prominent in subjects with a high aldosterone: renin ratio.

### Pulsewave analysis and velocity

In parallel with the detailed invasive assessment of endothelial function described above, a larger cohort of each sub-group undergoes a non-invasive measurement of vascular compliance and pulse waveform characteristics using the Sphygmocor technique. This measurement is likely to be influenced by the baseline ratio, and for this reason balanced numbers of subjects in each subset are recruited. This measurement will be made at the end of each treatment period.

### Exercise blood pressure

All subjects also have their blood pressure measured after a 3-minute exercise step-test, The Dundee Step Test. Preliminary data suggests that exercise BP is correlated with the aldosterone: renin ratio[25].

### Plasma and urinary steroids

A blood sample for plasma steroids is taken at baseline. A 24 h urine collection is also done and will be analysed for electrolytes, aldosterone and urinary steroids including 18-oxocortisol. These data will be used to investigate if there are any predictors of the BP response that are superior to the aldosterone: renin ratio.

### Plasma PIIINP (Procollagen Type III N-terminal Peptide)

Plasma PIIINP is a marker for collagen formation in the heart and spironolactone has been found to reduce PIIINP in heart failure[26]. We wish to examine whether if this is also the case in hypertension.

### DNA

A blood sample is drawn to assess the effect of polymorphic variation at the aldosterone synthase (CYP11B2) gene (SF1, 344T/C and intron 2 conversion) on baseline phenotypic abnormalities and response to therapy. In addition, all subjects are screened for the gene for glucocorticoid remediable aldosteronism (GRA).

### Ethical approval

The main study and sub-studies have ethical approval from Tayside Committee on Medical Research Ethics and West Ethics Committee, Western Infirmary, Glasgow.

## Discussion

The numerous deleterious effects of hypertension[27] dictate the need for a systematic approach for its treatment. In spite of various therapies, resistant hypertension is widely prevalent [28]. Among various factors, primary aldosteronism is an important cause of resistant hypertension and is now more commonly recognised[4,5,29]. Moreover even in treated hypertensives, the mortality remains up to five times higher compared to normotensives[30]. One of the factors might be inadequate blood pressure control due to usage of inappropriate drugs. More significantly, hypertensives with primary aldosteronism are also exposed to various other deleterious effects of excess aldosterone [31]. Aldosterone has deleterious effects on the myocardium and induces myocardial hypertrophy, fibrosis and necrosis[32,33]. Aldosterone is implicated in heart failure and its blockade in significantly reduces mortality[22,34]. Aldosterone also induces endothelial dysfunction[35]. Hence treating hypertension with specific aldosterone antagonists may be a better approach in this group of patients. It may lead on to better blood pressures with fewer medications[16].

## Competing interests

The author(s) declare that they have no competing interests.

## Authors' contributions

All authors are involved in design and acquisition of data, analysis and interpretation of data. They have been involved in drafting the manuscript and revising it critically for important intellectual content and have given final approval of the version to be published.

Prof Ian ford provides the main help with statistical analysis.

## Funding

This project is funded by a grant (number BA-01-25) awarded by Chief Scientist Office, Health Department, Scottish Executive. The funding was used by all authors in the collection of data, analysis, interpretation and publication of this manuscript.

## Pre-publication history

The pre-publication history for this paper can be accessed here:



## Figures and Tables

**Figure 1:**
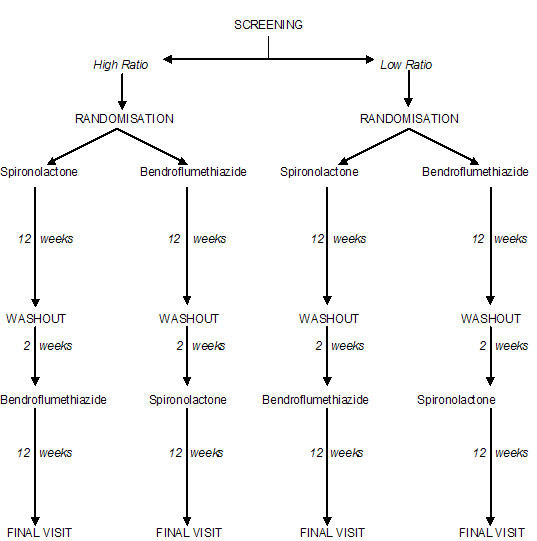
Renaldo Study Design.

**Figure 2:**
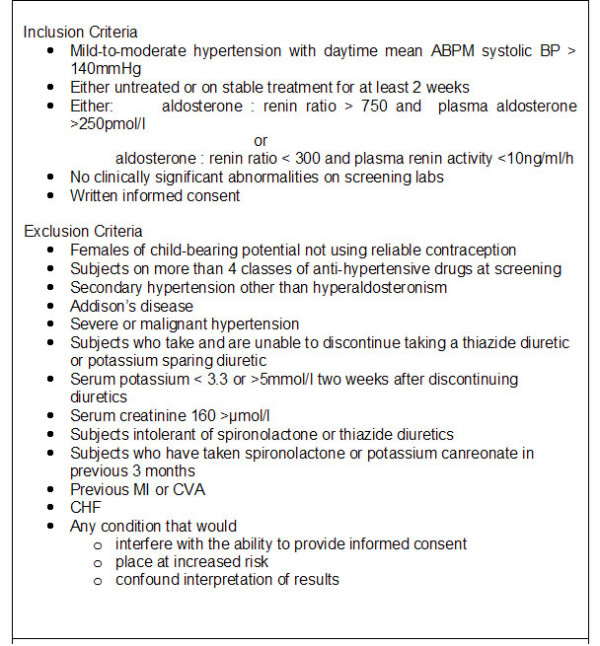
Inclusion/Exclusion criteria.
